# High Throughput Experimentation Using DESI-MS to Guide Continuous-Flow Synthesis

**DOI:** 10.1038/s41598-019-50638-7

**Published:** 2019-10-14

**Authors:** Bradley P. Loren, H. Samuel Ewan, Larisa Avramova, Christina R. Ferreira, Tiago J. P. Sobreira, Kathryn Yammine, Huiying Liao, R. Graham Cooks, David H. Thompson

**Affiliations:** Department of Chemistry, Purdue University, Purdue University Center for Cancer Research, Multi-disciplinary Cancer Research Facility, Bindley Bioscience Center, 1203 W. State Street, West Lafayette, IN 47907 USA

**Keywords:** Sustainability, Synthetic chemistry methodology

## Abstract

We demonstrate the use of accelerated reactions with desorption electrospray ionization mass spectrometry (DESI-MS) as a tool for predicting the outcome of microfluidic reactions. DESI-MS was employed as a high throughput experimentation tool to provide qualitative predictions of reaction outcomes, so that vast regions of chemical reactivity space may be more rapidly explored and areas of optimal efficiency identified. This work is part of a larger effort to accelerate reaction optimization to enable the rapid development of continuous-flow syntheses of small molecules in high yield. In order to build confidence in this approach, however, it is necessary to establish a robust predictive connection between reactions performed under analogous DESI-MS, batch, and microfluidic reaction conditions. In the present work, we explore the potential of high throughput DESI-MS experiments to identify trends in reactivity based on chemical structure, solvent, temperature, and stoichiometry that are consistent across these platforms. N-alkylation reactions were used as the test case due to their ease of reactant and product detection by electrospray ionization mass spectrometry (ESI-MS) and their great importance in API synthesis. While DESI-MS narrowed the scope of possibilities for reaction selection among some parameters such as solvent, others like stoichiometry and temperature still required further optimization under continuous synthesis conditions. DESI-MS high throughput experimentation (HTE) reaction evaluation significantly reduced the search space for flow chemistry optimization, thus representing a significant savings in time and materials to achieve a desired transformation with high efficiency.

## Introduction

Application of continuous-flow technologies has attracted the attention of chemists and the chemical and pharmaceutical industries in recent years due to desirable qualities compared to batch technologies such as improved safety, improved product quality environmental factor (E-factor), decreased inventory burden amenability to automation, reduced opportunities for human error compactness, and facile reactor reconfiguration^1–4^. Additionally, the superior mixing, improved heat and mass transfer, ease of scaling, and control over other process parameters in continuous-flow can lead to more efficient reactions and potentially even enable chemistries not possible in batch^1,2,5^. Additional advantages of continuous-flow technologies are their capacity for integration of reaction, purification, and analytics into a single compact automated system^6–9^. Although continuous-flow methods can enable the execution of efficient reactions in short residence times, the optimization process can still be costly and time consuming if many reagent types and conditions must be screened. Some laboratories have addressed this issue by integrating continuous-flow methods with on-line reaction monitoring to develop self-optimizing synthesis systems^10–12^. High throughput experimentation (HTE) also has tremendous potential to impact reaction selection, optimization, and discovery efforts^13–15^.

HTE is transforming the drug development process^16–19^. In much the same way that these tools have accelerated drug discovery, HTE in organic reaction exploration is poised to drastically boost the efficiency of reaction discovery, route selection, and step optimization. Several different approaches have been used to screen reactions in both batch^13,14^ and continuous-flow^11,15,20^ under high throughput conditions. One approach involves accelerated reaction screening in microdroplets to inform scaled up synthesis^6,21–26^. Our efforts are particularly focused on utilizing reaction acceleration to inform continuous-flow syntheses^6,21–23^. We have recently reported an extension of this approach to a high throughput format using desorption electrospray ionization mass spectrometry (DESI-MS)^27^. This system is capable of running several thousand reactions per hour in an automated fashion. We now report the application of this HTE method to guide the optimization of microfluidic reactions. Batch reactions in well plate arrays were used to examine the connection between reactions run with the DESI, batch, and continuous-flow reactors. Each combination of substrate, stoichiometry, and solvent was first screened with the high throughput DESI reactor. Reaction mixtures for DESI experiments were prepared with a Beckman Coulter Biomek FX liquid handling robot and subsequently transferred to a porous PTFE substrate using a magnetic pin tool to print 50 nL of each reaction mixture onto the substrate surface. This plate, with each reaction spot containing less than 1 µg of material, was then transferred to the mass spectrometer for DESI-MS imaging of the surface. As the plate is rastered beneath the solvent sprayer, the material on the surface is desorbed and secondary droplets are collected and rapidly analyzed by the mass spectrometer in a manner that has the potential to accelerate the reaction of interest^27^. The speed and compatibility of the HTE platform with automated DESI-MS analysis contrasts sharply with the traditional process of identifying preferred reaction conditions through an iterative series of batch experiments.

**Figure 1:**
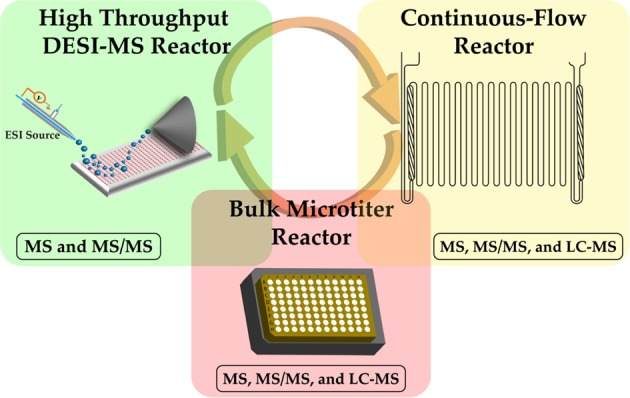
Conceptual representation of the reactor types and exchange of reactivity data between the (green) accelerated droplet reactor (red) bulk microtiter reactor and (yellow) continuous-flow reactor. Analytical tools used to monitor the reaction outcomes in each case are also shown.

The main strategy of this HTE approach is to utilize the highest throughput tool in the HTE system, DESI-MS, to produce an initial reactivity heat map, by-product profiles, and structural analyses of the reaction outcomes via MS/MS^27^. The remarkable throughput of this system is enabled by the robotic preparation of reaction arrays at high densities and by acceleration of the reactions themselves in thin films or in charged microdroplets^25,27^. Whether each reaction is accelerated in a thin film or a microdroplet or is simply occurring after initial mixing remains undetermined, but the utility of the technique overall is not dependent on this distinction and is thus not the focus of this work. Once the heat map has been produced, reaction conditions corresponding to the “yes” areas of the map may then be elevated to the next level of HTE, while the “no” conditions are abandoned. A more limited range of “yes” conditions can then be tested in small volume batch reactions prepared in microtiter plates as an intermediate step to provide more textured data with respect to temperature effects and product quantitation. Information from smaller more focused screens in bulk microtiter and continuous flow reactors may both serve as a validation of the DESI-MS results, as well as serving to refine subsequent DESI-MS HTE (Fig. 1). Data from this series of experiments can then be used to guide reaction optimization to achieve the highest yielding transformations under continuous-flow conditions in an efficient manner. The “no” conditions that are abandoned in this way represent a significant reduction in manpower and material waste, as the need to review those conditions in continuous flow or batch experiments is eliminated.

In order to build the necessary confidence in this heat map-based reaction pruning approach, it is important to demonstrate that reactivity trends are consistent across each platform. Since the goal of this HTE strategy is to guide downstream optimization and scaling by narrowing down the potential reaction space, it is not essential to quantitatively determine the reaction outcomes in the DESI-MS experiment. The focus instead is on identifying the presence or absence of target compounds and/or reaction byproducts in a rapid “yes/no” evaluation for subsequent optimization and scaling in flow.

N-alkylation reactions were chosen as the test case for this system due to their high relevance in medicinal chemistry^28^. Trends in reactivity were explored in each reactor type with varying substrate, solvent, stoichiometry and temperature (the latter only in the cases of bulk microtiter and continuous-flow). Our first experiment evaluated aniline reactivity as a function of varying electron demand substituents using a common electrophile, benzyl bromide. Once an initial correlation between the screening approaches was established, we expanded the scope of amine types using both the high throughput DESI and continuous-flow experiments. Scale up of a reaction in continuous-flow based on the outcome of this larger experiment was also demonstrated.

## Results

### Aniline reactivity screen

**Figure 2:**
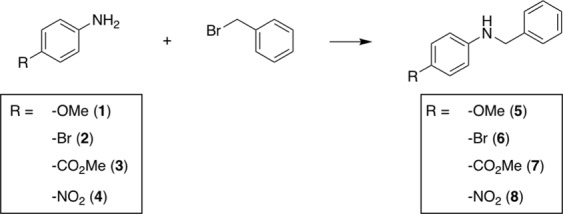
N-alkylation reactions executed on the DESI, batch microtiter, and continuous-flow reactor platforms.

We began our search of reactivity trends using four anilines having para-substituents that imposed varying electronic demand on the amine nucleophile (Fig. 2). It is well known that aniline nucleophilicity is strongly influenced by substituents that alter the electron density in the aromatic ring. We chose this transformation as a test case to establish whether the expected reactivity trends would be consistent across each reactor platform. Each aniline was mixed with benzyl bromide in three solvents (ACN, Toluene, DMSO) at three stoichiometries (10:1, 1:1, 1:10). Observation of the expected trends across the different reactor platforms would serve as the first indication that DESI and/or batch pre-screening may offer valid predictions for the corresponding continuous-flow reaction outcomes.

The high throughput DESI experiment was carried out by preparing reaction mixtures in standard polypropylene 384-well plates using a Beckman Coulter Biomek FX liquid handling robot. A magnetic pin tool with 50 nL slotted pins was then interfaced with the constant volume transfer arm of the robot to deposit a small volume of each reaction mixture onto a porous PTFE substrate. This plate, with each spot representing a unique set of reaction conditions, was then transferred to the DESI-MS for analysis immediately following its preparation. In a separate experiment, 50 uL of, the same set of conditions, rather than being spotted onto the porous PTFE plate, were deposited into glass vials inserted within 96-well aluminum blocks for high throughput batch microtiter reaction screening. These reaction blocks were compression sealed with a PFA-lined silicone mat, allowing the reactions to be heated. Four such plates were prepared from a master plate and heated at 50, 100, 150, and 200 °C, respectively, for 30 minutes, followed by dilution into ACN to quench the reactions prior to storage at −80 °C for subsequent analysis by ESI-MS. The continuous-flow experiments were executed in 10 µL glass reactor chips mounted on a Chemtrix Labtrix S1 reactor system. The flow reactions were similarly quenched by dilution into ACN and storing at −80 °C until ESI-MS analysis. Since the goal of this initial experiment was to track reactivity trends rather than optimize individual transformations, the continuous-flow experiments were carried out with a constant residence time of 30 sec. Quantitation of the batch and continuous-flow reactions was conducted using LC-MS and referenced to an external calibration curve to mitigate concerns that the observed conversion trends might be dependent on the varying ionization efficiencies of the anilines.

**Figure 3:**
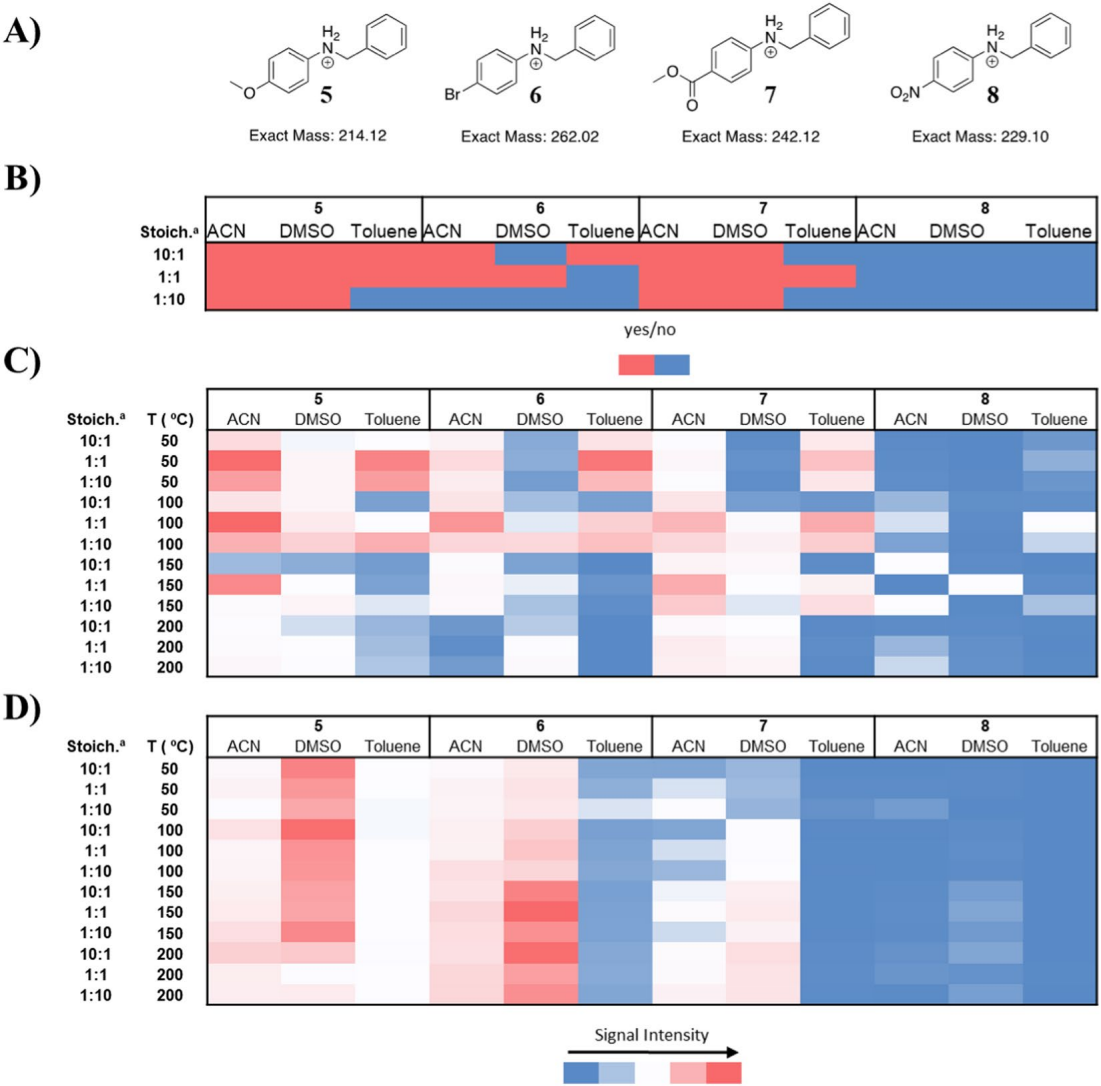
(**A**) Structures of the reaction products. Heat map representations of **(B**) yes/no reaction outcomes based on ion intensities in the high throughput DESI-MS experiment, (**C**) LC/MS quantitation of the batch experiment, and (**D**) LC/MS quantitation of the continuous-flow experiment. ^a^Stoichiometries are reported as ratios of aniline:benzyl bromide. The raw MS intensity data for each reaction appear in the Supplemental Information (Fig. S1).

Heat map representations of all three experiments, based on product ion intensities for the DESI experiment and LC/MS determined concentrations for the batch microtiter and continuous-flow experiments, are shown in Fig. 3. The color scale for each of these heat maps is set to the same range of signal intensities across the entire heat map. For the DESI heat map, a binary “yes/no” scale was used with a threshold of 30 ion counts for the target mass to charge ratio (m/z), while the other heat maps are shown as a concentration-based color gradients. The high throughput DESI-MS experiment (Fig. 3B) was conducted at a 1,536 well plate density using 50 nL transfer pins to deposit the sample onto a porous PTFE plate that was cut to the dimensions of a standard microscope slide. This experiment showed that p-anisidine (**1**) was readily mono-alkylated, whereas p-bromobenzene (**2**) and methyl 4-aminobenzoate (**3**) had some conversion to product, and p-nitroaniline (**4**) produced minimal monoalkylation product. Further, there appears to be a strong solvent effect in the case of **3**, with ACN and DMSO favoring product formation, whereas toluene failed to produce a significant amount of product. These same substrate and solvent trends were observed in the continuous-flow experiments (Fig. 3D). It is notable that these trends appear to be consistent whether the continuous-flow data was analyzed qualitatively (ESI-MS) or quantitatively (LC-MS), suggesting that the observed conversion trend was not merely an artifact of ionization efficiency (Fig. S1). This important finding suggests that DESI-MS may be a useful HTE tool for reaction optimization.

While the substrate and solvent trends appear to translate well for reactions run under DESI and flow conditions, some disagreement between the batch and flow results were noted. This was particularly true when considering the reactivity trends as a function of temperature. The continuous-flow reactions showed a strong temperature dependence, wherein higher temperatures provided higher yields, while lower temperatures were more favorable under batch conditions. We attribute these findings to the longer reaction times used in the batch experiment (30 min), resulting in thermal degradation of the products and/or starting materials when heated to 150 or 200 °C. To further probe this, a series of continuous-flow experiments were executed at 200 °C using an 8 minute residence time. Comparison of the full mass spectra for these flow experiments and batch experiments showed significantly reduced product formation relative to the shorter timeframe flow reactions. We infer from these findings that thermal degradation was the likely source of ineffective data translation between the batch microtiter and continuous-flow experiments (Fig. S2). These observations also highlight an inherent limitation in batch HTE. Even though the aluminum blocks enable heating of the reaction arrays at reflux, heat transfer is much slower and less well controlled in these reactors compared to continuous-flow reactors. This, in turn, leads to less control over reaction conditions, resulting in the thermal degradation that was observed in the mass spectra.

In spite of this complication, the reactivity trend fidelity observed using the DESI and continuous-flow reactors suggests that DESI-MS HTE represents a path for dramatically increasing the rate of reaction optimization. Although the DESI reactor is not able to heat reactions, the ultimate goal of the DESI experiment is to deploy its thousands-of-reactions per hour throughput to rapidly scout out a vast chemical reactivity space to identify a significantly smaller number of reactions that can then be carried forward for optimization and scaling.

### Expanded N-alkylation screen

**Figure 4:**
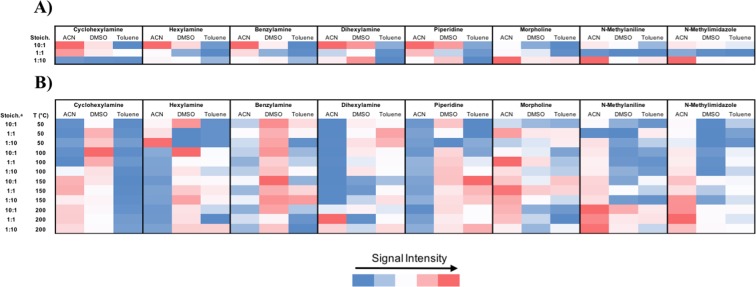
Heat map representations of N-alkylation products observed using various amine nucleophiles. (**A**) ion intensities in the high throughput DESI experiment and (**B**) ion intensities in the continuous-flow experiment. ^a^Stoichiometries are reported as ratios of amine:benzyl bromide. *Bold outlines indicate regions of the heat map where the conditional color formatting was set. The raw intensity data for each reaction appears in the Supplemental Information (Fig. S3).

The aniline experiment established a correlation between the reaction outcomes in high throughput DESI and continuous-flow reactors, thereby confirming the utility of DESI as a predictive tool for judging the likely outcome of flow experiments. These encouraging results led us to expand the scope of amine substrates to determine whether the correlations would hold for a more diverse family of amines. Their reactivity was surveyed using DESI and continuous-flow to determine whether the observed reactivity trends would translate well across these reaction platforms platforms. Given the degradation complications observed with the previous batch microtiter experiments, this reactor was not utilized in the expanded N-alkylation experiments. Each combination of substrate, stoichiometry, and solvent was again screened under the same DESI conditions as in the previous experiments, except that a PTFE plate the size of a standard well plate was used. The reactions in this case were spotted in quadruplicate using 50 nL slotted pins to produce a plate at standard 1,536 well density. Rhodamine was incorporated on the plate as a fiducial marker to allow in-house developed software to determine the exact x and y coordinates for each reaction spot. Heat maps were again generated for both the DESI-MS and continuous-flow datasets (Fig. 4). The heat maps for this dataset are based on ion intensities and organized by the different amine starting materials. Conditional color formatting was reset for each amine to avoid a misinterpretation of the heat maps due to trends based on differences in ionization efficiency rather than chemical reactivity. These DESI heat maps are displayed as a multi-color scale in the same manner as the flow reaction heat map. This is because with the color gradient set for each amine individually, the threshold for a yes/no is different for each amine. Since we are not comparing different substrates to each other in this experiment, we are able to more easily observe any solvent and stoichiometry trends for each individual amine in this way. Given that the main objective of this experiment was to streamline the optimization of any one of these reactions, reporting the data in this manner most clearly illustrates the high throughput potential of the method. The raw intensity data for each reaction appears in the Supplemental Information (Fig. S3).

The DESI results again suggest that ACN is the best solvent in most cases, while DMSO will generally be superior to toluene for these reactions. Although this solvent trend did not precisely track across all of the continuous-flow reactions, it translated well overall. Specifically, ACN was the hottest spot in the continuous-flow experiments for 5 out of 8 amines and DMSO was the hottest spot for 2 out of 8 amines. Piperidine was the only case where toluene was the hottest spot in the continuous-flow dataset. Although piperidine is an example of a case where the DESI and continuous-flow experiments are misaligned, the DESI-MS results indicated a lower overall ion intensity in all of its reactions compared to the other amines (Fig. S3). Therefore, while the reactivity trends across the solvents did not appear to fully translate in this case, the DESI-MS results did reveal that the overall reactivity of piperidine was lower than the other amines. The likelihood that this is the source of the inconsistency is supported by the finding that significant product formation in this reaction only occurred at elevated temperature in continuous-flow.

In addition to rapidly generating reactivity heat maps, the high throughput DESI-MS platform is also able to acquire MS/MS data for the structural confirmation of products and by-products. This capability is demonstrated in the Supporting Information (Fig. S4) for the reaction between cyclohexylamine and benzyl bromide where an MS/MS spectrum for this reaction from the high throughput DESI-MS experiment as well as an MS/MS spectrum from the continuous-flow synthesis of the same product are shown. The protocol for the MS/MS experiment has been previously reported^27^. In brief, the x,y coordinates of the reaction hits are generated using in-house software. This data can then be used to inform the 2D DESI-MS source to return to those spots and generate MS/MS data immediately following full-scan data acquisition and processing.

### Using HTE to scale a reaction

Since our ultimate goal was the selection from a large set of reaction outcomes a smaller and more manageable set of optimal conditions for scale up, we chose a single substrate, cyclohexylamine, to test the entire HTE workflow from DESI-MS to scalable product formation in continuous-flow. The DESI experiment showed ACN as the optimal solvent, with DMSO as second best. In this case, the DESI-MS results were validated in the previous experiment, showing no false positives or false negatives in the DESI to flow prediction. Based on these experiments, a focused set of microfluidic reactions were performed in ACN and DMSO with varying residence times at an increased concentration of 1 M to better reflect continuous production conditions. The ACN reaction in continuous-flow at this concentration, however, was a practical failure due to precipitation and clogging of the reactor chip by the monoalkylated product at this increased concentration, thus leaving DMSO as the only viable option for optimizing production in continuous-flow at this concentration (Table 1). In this case, we were able to reduce the search by one third of possible conditions in our predefined chemical space. In the large data sets that this system is designed for, such a reduction in search space may represent a significant time savings and reduction in chemical waste generated. The yield of these reactions was determined by LC-MS quantitation, with the highest yields occurring at a residence time of 30 seconds and a temperature of 100 °C, translating to 56.6% yield and a production rate of 42.8 mg/hr.

**Table 1:** Optimization of the reaction between cyclohexylamine and benzyl bromide.

Entry	R_T_ (sec)	Stoich.^a^	Temp. (°C)	Yield (%)
1	15	1:2	100	29.8
2	15	1:1	100	34.3
3	15	2:1	100	50.8
4	30	1:2	100	56.6
5	30	1:1	100	47.1
6	30	2:1	100	28.6
7	60	1:2	100	24.3
8	60	1:1	100	29.2
9	60	2:1	100	43.2

^a^Stoichiometries are reported as ratios of amine:benzyl bromide.

## Discussion

The utility of DESI-MS as an HTE tool for rapidly evaluating large numbers of reactions to help guide condition selection and optimization for continuous-flow synthesis was demonstrated. Although this HTE system is not capable of quantitatively determining the optimal condition for scale-up, it is able to narrow down the number of conditions that need to be evaluated during scaling efforts. A series of experiments were performed to test how well reactivity trends observed using the DESI and batch microtiter reactors can be used to identify the best synthesis conditions for use in a continuous-flow reactor. N-alkylation reactions were used as the test bed for these experiments, first using a focused screen of anilines having varied electron demand on the aromatic ring. This experiment provided encouraging evidence that reactivity trends observed as a function of solvent in the accelerated DESI reactor were able to translate well to continuous-flow reaction conditions. Translation of data from the batch microtiter in this case was less effective, but this appears to be due to degradation of material at the highest temperatures screened. While this represented a limitation of the batch microtiter reactor platform under the conditions employed, it is still a valuable tool for examining reactions run under bulk conditions that are commonly found in the literature.

DESI and continuous-flow reactions on a broader scope of amines were then performed. These experiments established that the reactivity trends observed with the aniline experiments also translated well to a more structurally diverse set of amines. While the reactivity heat map of each individual reaction did not completely track across the two platforms, the general trend of more polar solvents being preferred did translate. This is an important point since the goal of the DESI reaction platform is not to provide a single reaction condition that will be executed at scale, but rather to take an extremely large chemical reaction space and narrow it to something that can be more manageably evaluated in continuous-flow to discover the optimal conditions needed for scaling. To demonstrate this concept, a single amine, cyclohexylamine, was chosen for optimization and scale up in continuous-flow. The flow reaction optimization process resulted in a method capable of producing a 56.6% yield of the benzylated product at a residence time of 30 sec and an amine:benzyl bromide ratio of 1:2, representing a production rate of 42.8 mg/hr in a 10 µL reactor chip. In implementing our DESI-MS guided reaction selection model, we reduced the search space for the optimized reaction by one third. As our system develops and data sets grow, such a reduction in search space may represent a significant savings in time, energy, and chemical waste.

This study demonstrates the potential of high throughput reaction screening with DESI-MS to accelerate route selection and optimization. While we found DESI-MS to narrow the scope of possibilities for reaction selection based on solvent type, other parameters like stoichiometry like stoichiometry and temperature still required screening in a microfluidic reactor for optimization. While it has been demonstrated as useful in the translation of high throughput data to continuous-flow data in the case of N-alkylation reactions, in order to more fully establish its usefulness as a screening tool for organic reactions, the scope of chemistry conducted with this platform must be further expanded. Efforts devoted to the expansion of HTE throughput and refinement of the hardware and software for the automation of both reaction preparation and analysis are important next steps that are ongoing.

## Methods

### General procedure for DESI-MS experiments

The DESI-MS experiments were conducted as described previously^27^. A Biomek FX liquid handling robot (Beckman Coulter) was used to distribute reaction mixtures into master well plates (either 96 or 384 well plates, depending on the scope of the experiment). The 96-tip transfer pod (pod1) and the Span-8 transfer pod (pod2) were both utilized to generate the target set of reaction conditions. The final reaction concentration for all DESI-MS experiments was 50 mM (20–30 µL in the final 384 well master plate). A magnetic pin tool (V&P Scientific, Inc.) was interfaced with pod1 of the liquid handling robot and used to transfer 50 nL volumes of reaction mixtures from the master well plate to the porous PTFE surface for DESI-MS. A commercial DESI source (Prosolia, Inc.) and a Thermo LTQ linear ion trap were utilized to execute the DESI-MS experiment. Experiments were conducted in positive-ion mode (m/z 50–500) with pure methanol as the spray solvent (3 µL/min). Parameters for the mass spectrometer and speed of the DESI stage were optimized previously^27^. In-house software was used to process the data and generate spreadsheets from which heat maps were prepared.

### General procedure for qualitative MS analysis

Mass spectral analysis was performed for each flow and batch reaction sample using a Thermo TSQ triple quadrupole mass spectrometer (Thermo Fisher Scientific) equipped with an autosampler and electrospray (ESI) ionization. Reaction samples were diluted 1:1000 into ACN upon collection and cooled to −80 °C to quench the reactions before warming to room temperature immediately prior to analysis. The distance between the tip of the spray emitter and the ion transfer capillary to the MS was kept constant at ca. 1.5 cm. Experiments were performed using a Thermo-Fisher HESI-II probe and Ion Max ion source. A spray voltage of 3.5 kV was used for all analyses. Positive-ion mode was used for all analyses.

### Quantitative LC/MS analysis

Quantitative measurements were carried out with an Agilent 6460 triple quadrupole LC/MS with an Agilent Series 1200 degasser, binary pump, and autosampler. An XBridge Phenyl 3.5 µm 2.1 × 100 mm column was used to separate the aniline products, while an Atlantis dC18 3 µm 2.1 × 150 mm column was used to separate the cyclohexylamine products. The elution used a gradient of ACN:H_2_O with 0.1% formic acid. Calibration curves were made using standards of the respective products.

### General procedure for bulk microtiter experiments

A Biomek FX liquid handling robot was used to distribute reaction mixtures into aluminum well plates fitted with glass vials (Analytical Sales & Services, Inc.). A master plate was prepared with varying substrates, solvents, and stoichiometries and then distributed into four identical plates that were sealed with a PFA film and two silicone rubber mats before heating for 30 min at 50, 100, 150, and 200 °C, respectively. Each reaction vial was prepared at 50 mM and contained 50 µL of reaction mixture. After allowing the plates to cool, the Biomek FX was used to dilute the reaction mixtures to 500 µM in ACN. A Thermo TSQ mass spectrometer equipped with an autosampler was used for the qualitative analysis of these reactions. The protocol for quantitative analysis is described above.

### General protocol for continuous-flow experiments

All microfluidic reactions were carried out using a Chemtrix Labtrix S1 system equipped with 3223 glass reactor chips. Solutions of 0.05 M amines and 0.05 M benzyl bromide in toluene, ACN and DMSO were prepared. Syringes were loaded with each of these solutions and positioned on the first two inlets of a 10 μL Chemtrix 3223 chip. The reactants were engaged with 30 s residence times at temperatures of 50, 100, 150, and 200 °C. Samples were collected and immediately diluted 1:1000 in ACN and stored at −80 °C prior to analysis.

### Continuous-flow degradation experiments

Reactions were carried out using a Chemtrix Labtrix S1 system equipped with 3223 glass reactor chips as described above, except that the reactants were flowed with 8 minutes residence times at 200 °C. Samples were then handled as described above.

### Continuous-flow optimization experiments

Reactions were carried out using a Chemtrix Labtrix S1 system equipped with 3223 glass reactor chips as described above, except that solutions of 1 M amine and 1 M benzyl bromide in toluene, ACN and DMSO were used. The reactants were flowed with 15, 30, and 60 s residence times at temperatures of 100 °C before collecting and handling as described above.

## Supplementary information

DESI HTE to Flow N-alkylation Supplementary Information
