# Supplementary material for: High Throughput Experimentation Using DESI-MS to Guide Continuous-Flow Synthesis
Source: Sci Rep. 2019 Oct 14;9:14745. doi: 10.1038/s41598-019-50638-7 (PMC6791872; doi:10.1038/s41598-019-50638-7)
Supplement: Supplementary file 1 — DESI HTE to Flow N-alkylation Supplementary Information [file 41598_2019_50638_MOESM1_ESM.pdf]

SUPPORTING INFORMATION

High Throughput Experimentation Using DESI-MS to Guide Continuous-Flow Synthesis

Bradley P. Loren‡, H. Samuel Ewan‡, Larisa Avramova, Christina R. Ferreira, Tiago J. P. Sobreira, Kathryn Yammine, Huiying Liao, R. Graham Cooks*, and David H. Thompson*

Department of Chemistry, Purdue University, Purdue University Center for Cancer Research, Multi-disciplinary Cancer Research Facility, Bindley Bioscience Center, 1203 W. State Street, West Lafayette, IN, 47907 USA

‡These authors contributed equally to this work. Correspondence to: davethom@purdue.edu

Content **Page Number**

1. Figure S1: Aniline experiment heat maps including ion intensity or concentration values. **SI-2**
2. Figure S2: Elevated temperature leads to degradation. **SI-4**
3. Figure S3: Expanded substrate scope experiment heat maps including ion intensity values. **SI-8**

Figure S1: Aniline experiment heat maps including ion intensity or concentration values.

DESI raw intensity (arbitrary units of ion intensity).

	1			2			3			4		
	ACN	DMSO	Tol.	ACN	DMSO	Tol.	ACN	DMSO	Tol.	ACN	DMSO	Tol.
**10:1**	331	323	393	83	7	76	101	78	28	0	0	0
**1:1**	285	232	373	217	118	16	124	187	37	0	0	0
**1:10**	144	76	15	25	24	2	49	35	2	0	0	0

Quantitative measurement of microfluidic reactions (concentrations in ng/mL).

		1			2			3			4		
Eq BnBr	T (C)	ACN	DMSO	Toluene	ACN	DMSO	Toluene	ACN	DMSO	Toluene	ACN	DMSO	Toluene
10	50	158.469983	4844.17112	99.1007196	642.5156	1308.952	80.47918	166.2872	38.14806	7.112387	16.33531	0.231241	0.168573
2	50	95.8823726	1482.23354	56.6474077	356.0102	844.3161	39.06616	44.95545	28.57748	2.660867	5.175886	0.192303	0.083527
1	50	689.737789	5751.95349	138.315508	680.1945	1513.745	33.49316	79.37003	43.79325	2.148461	2.486858	1.787329	0.551062
0.2	50	869.832449	6208.0616	129.915865	435.6533	1504.527	30.56399	37.59223	48.23396	1.79467	1.630832	1.435623	1.198635
0.01	50	403.428982	6896.29623	105.470099	376.3541	1214.863	25.4829	24.67513	40.34876	1.058441	1.264997	1.486857	0.093119
10	100	633.16582	5863.6261	116.223011	1761.31	2309.867	26.66675	41.28754	135.6073	0.962355	1.129567	2.652392	0.509708
2	100	226.579816	1413.55442	58.9427555	1132.011	1880.449	19.30594	61.86592	121.5461	0.922828	0.935354	2.501281	0.412333
1	100	596.553399	6037.69965	114.632218	769.5317	3231.669	23.66905	76.04906	212.9958	1.067931	1.14451	4.360998	0.321272
0.2	100	612.598286	9286.32959	137.079886	399.0764	3477.009	22.90528	51.85222	227.875	1.215638	1.543855	3.750885	0.518536
0.01	100	1657.14961	8090.40818	98.7499063	866.3824	2620.109	20.51092	24.59878	167.2782	0.724992	0.921845	3.063027	0.658055
10	150	1790.72828	6598.46552	135.388782	1881.368	6194.111	22.17251	72.97173	755.4033	0.906812	2.848365	14.80027	0.111557
2	150	677.013161	1282.67885	76.9626852	1302.967	4873.804	16.46414	88.25759	652.7673	0.947956	1.557033	14.8822	0.484368
1	150	1108.40146	5012.54724	152.198524	2202.054	8326.325	21.80124	262.0943	1135.943	1.271164	3.325924	25.03769	0.339782
0.2	150	1910.97132	5796.83413	160.269731	1389.623	8995.261	23.98034	128.1214	1308.938	1.403523	5.022542	25.53068	0.591859
0.01	150	881.858962	5158.32796	131.169479	1500.71	6920.384	20.01145	94.80985	919.3535	0.998882	3.185632	18.58315	0.516301
10	200	969.703101	1081.70257	156.370688	2275.508	6204.278	25.63708	732.51	1461.639	2.110739	1.528041	19.33769	0.720862
2	200	478.900588	117.855338	69.3666183	788.0994	1523.825	19.38505	190.3803	491.6776	1.773237	5.509507	3.434077	0.859371
1	200	991.691428	165.23234	149.425621	2216.848	5327.382	29.07996	284.4254	1551.532	3.320357	10.72172	7.383881	1.016854
0.2	200	1244.46937	2005.25162	177.16073	1402.184	8968.588	31.14907	309.3722	2247.038	3.467545	11.10985	19.64135	0.202862
0.01	200	2599.28591	3014.44314	187.792895	1733.014	8085.147	27.70413	269.3137	1835.813	2.66926	7.063528	25.07077	0.363045

Qualitative measurement of microfluidic reactions (arbitrary units of ion intensity).

		1			2			3			4		
Eq BnBr	T (C)	ACN	DMSO	Toluene	ACN	DMSO	Toluene	ACN	DMSO	Toluene	ACN	DMSO	Toluene
10	50	4228490	20178802	2657457	3257105	538492	1436432	3580453	387004.4	127685.8	346091.2	952716.4	64595.94
2	50	2706137	3427214	1761194	1897551	344756.1	628082.5	2935999	391564.5	91923.27	869813.3	408211.5	15547.94
1	50	12628953	19454960	3381857	2277050	220357.2	1052345	8910785	421253.4	126330.9	460239.6	576109.9	65678.08
0.2	50	4694628	21735205	1339869	2911265	212204.2	780014.4	2838396	190618.3	156442.1	537589.1	711645.5	48494.58
0.01	50	728796.1	14836135	2252734	1202560	101185	1640901	2136918	262664.4	200994.2	199225.2	726757.1	140263.3
10	100	1767412	15875771	2359612	5991086	489741.5	1150274	5280434	351368.9	141875.1	822317	504837.6	125999.3
2	100	3869670	9474064	1343317	10910710	234946.3	670211	14687378	303931.2	81410.18	384800	727218.7	53051.6
1	100	8031471	17485714	2141656	3712089	685521.3	437506.9	10428368	338654.9	126633.4	1072777	631393.5	17090.45
0.2	100	4532933	26436641	1313773	2177286	676113.9	1911967	10591043	373529.3	242266.5	1583308	369429.2	66735.92
0.01	100	5045726	30964267	1363529	2845472	398102.5	2652179	2223890	548681.1	98345.58	758807.9	329932.8	30712.84
10	150	29914972	11541102	3045537	7744675	1180066	791962.8	1078363	691196.2	972303.6	797666.6	331683.5	68446.83
2	150	2430579	17383367	1585896	5452554	458390.9	441799.4	13840284	1058718	219290	1138793	416281	99663.91
1	150	31321623	12212533	3291822	7577208	582496.1	1758139	3201685	2444952	295358.1	1216875	199320.8	213916
0.2	150	20822880	30802387	2836183	2717132	1183498	1042558	3855388	2661850	170422.7	697362.7	453390.8	83929.63
0.01	150	8759174	14528701	3765829	2517350	1176249	3005840	4584639	1078310	143380.3	593520.4	440964.8	231457.5
10	200	21425675	13733143	6162209	8186811	545197.2	1105614	8304550	1675570	113475.4	712085.3	126578.6	119939.9
2	200	15332326	437442.4	1693204	3322588	247845.9	167362.6	4331793	1606522	122034.4	440681	271573.7	48905.83
1	200	38394933	1659858	3164515	5223471	832763.9	1014938	8207966	1335921	222331.5	906372.2	331240.9	56435.84
0.2	200	68752970	22231329	4519315	1706390	583745.6	797734.8	2296307	1203199	381623.1	1144066	406948.1	137648.2
0.01	200	20481602	15999247	5213342	3598431	969509.6	1790871	2027613	1767333	184462.3	500570.1	518565.7	41527.56

Quantitative measurement of batch reactions (concentrations in ng/mL).

		1			2			3			4		
Eq BnBr	T (C)	ACN	DMSO	Toluene	ACN	DMSO	Toluene	ACN	DMSO	Toluene	ACN	DMSO	Toluene
10	50	4436.84744	480.600357	4521.79681	833.8507	12.07352	3222.506	133.5336	2.859222	1127.766	2.770422	0.640258	7.454427
1	50	6785.15323	434.971391	5672.47216	1683.388	21.49311	6270.91	353.4821	5.140943	2820.139	2.11918	0.150058	23.09769
0.01	50	1612.92807	66.2137542	73.2110365	568.0986	19.89416	1251.377	127.7324	2.334681	956.1623	1.024803	0.389975	8.886231
10	100	3581.77755	2078.89974	3680.96643	1864.19	1764.764	2894.645	1867.978	591.4846	2270.6	15.1924	0.612834	46.25055
1	100	6897.84898	940.434796	93.3612739	4878.44	58.95525	2118.788	3338.068	235.2084	3874.595	52.98827	1.903576	91.69564
0.01	100	1201.96611	453.537798	14.4616671	1227.421	32.2707	13.57451	1158.858	12.49403	7.910045	27.95332	3.323347	4.147682
10	150	201.85213	445.177002	57.034856	277.7758	33.89862	2.654186	2398.968	57.23254	1527.17	82.27251	0.918018	34.15006
1	150	5431.4688	80.9946493	15.112334	310.5545	62.02034	6.686657	3808.312	99.13845	625.6473	0.169629	100.8731	2.90579
0.01	150	29.8082823	21.7107388	11.5048871	178.7811	15.47313	0.134448	506.7717	310.2142	2.12647	76.02461	1.381631	0.073483
10	200	367.591682	140.908488	37.0267691	10.9282553	213.657807	0.36791435	722.4187	455.2158	1.041835	48.0211	4.422044	0.92534
1	200	167.237859	84.9294628	31.6351588	2.87371251	135.99742	0.24086837	844.8222	327.3681	2.262354	27.19554	4.290544	0.245105
0.01	200	121.380273	51.694749	27.1828728	8.39224511	40.2597151	0.21140977	268.5793	88.17656	0.554521	2.076033	1.690316	0.88116

Figure S2: Elevated temperature leads to degradation.

Full Scan MS of high temperature batch reactions between p-anisidine and benzyl bromide.

3 = A1, ACN, 1:1, 200C
9 = A1, Toluene, 1:1, 200C
15 = A1, DMSO, 1:1, 200C

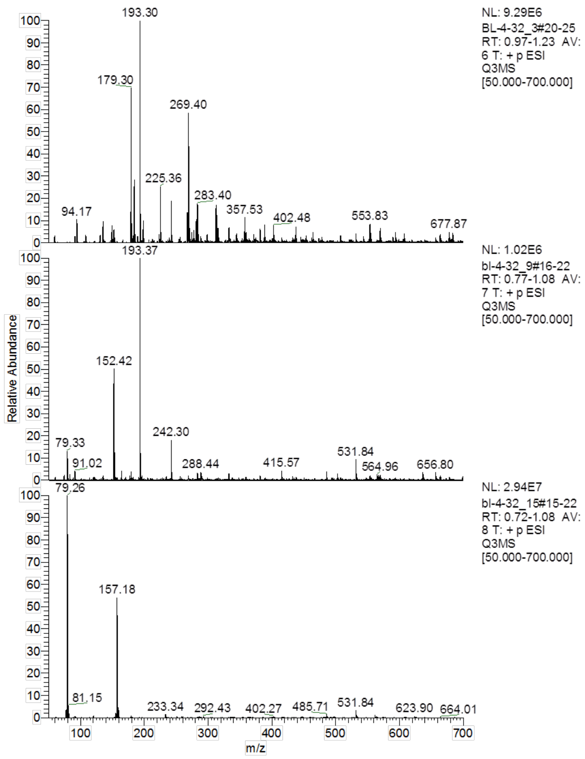

Full Scan MS of high temperature batch reactions between p-bromoaniline and benzyl bromide.

21 = A2, ACN, 1:1, 200C
49 = A2, Toluene, 1:1, 200C
55 = A2, DMSO, 1:1, 200C

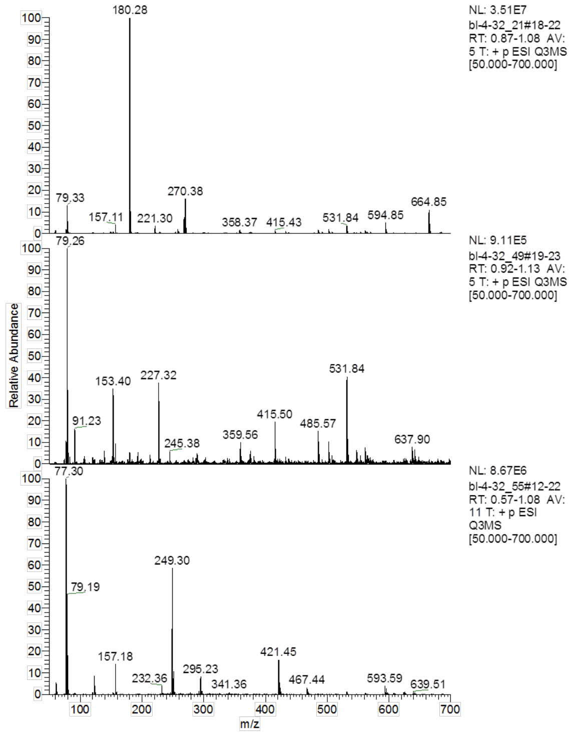

Full Scan MS of high temperature batch reactions between 4-methylaminobenzoate and benzyl bromide.

61 = A3, ACN, 1:1, 200C
89 = A3, Toluene, 1:1, 200C
95 = A3, DMSO, 1:1, 200C

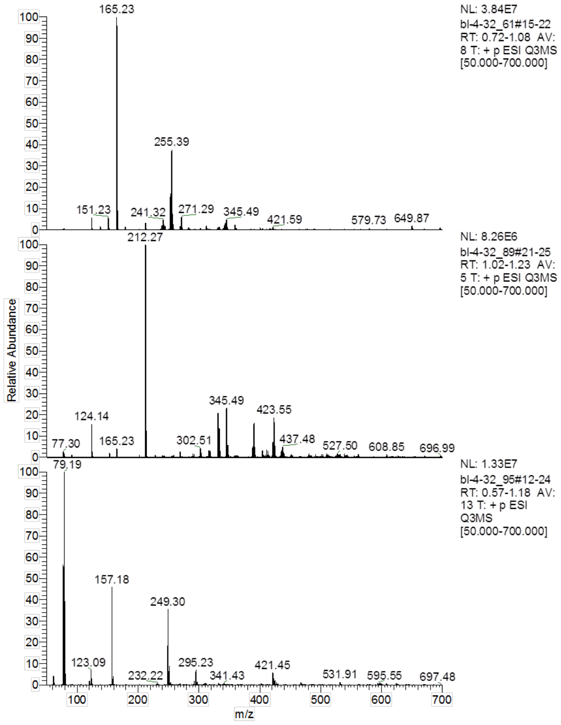

Full Scan MS of high temperature batch reactions between p-nitroaniline and benzyl bromide.

101 = A4, ACN, 1:1, 200C
107 = A4, Toluene, 1:1, 200C
135 = A4, DMSO, 1:1, 200C

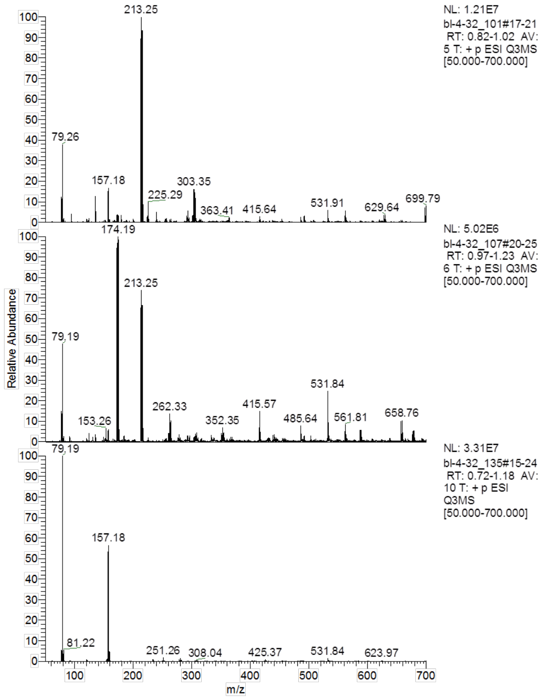

Full Scan MS of p-anisidine and benzyl bromide reaction in ACN at 200 ˚C. Top T_r = 30 sec, Bottom T_r = 8 min.

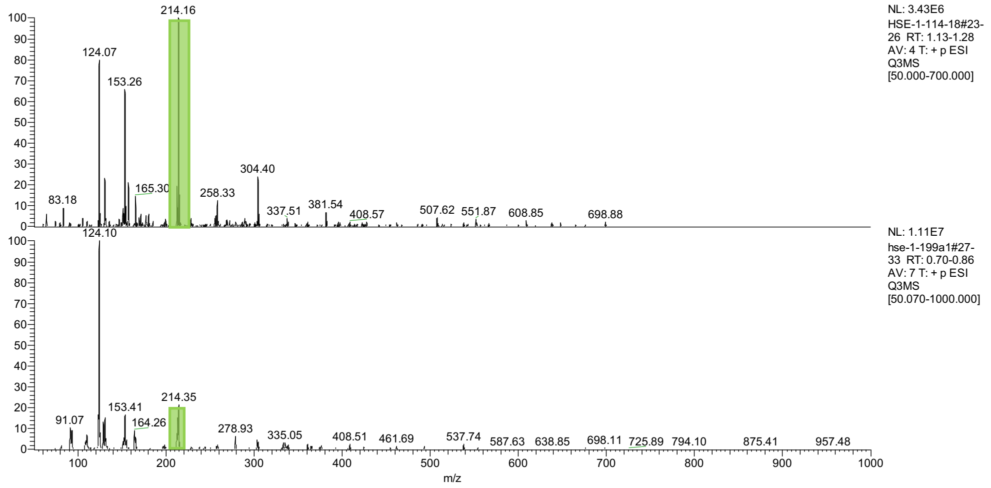

Full Scan MS of p-bromoaniline and benzyl bromide reaction in ACN at 200 ˚C. Top T_r = 30 sec, Bottom T_r = 8 min.

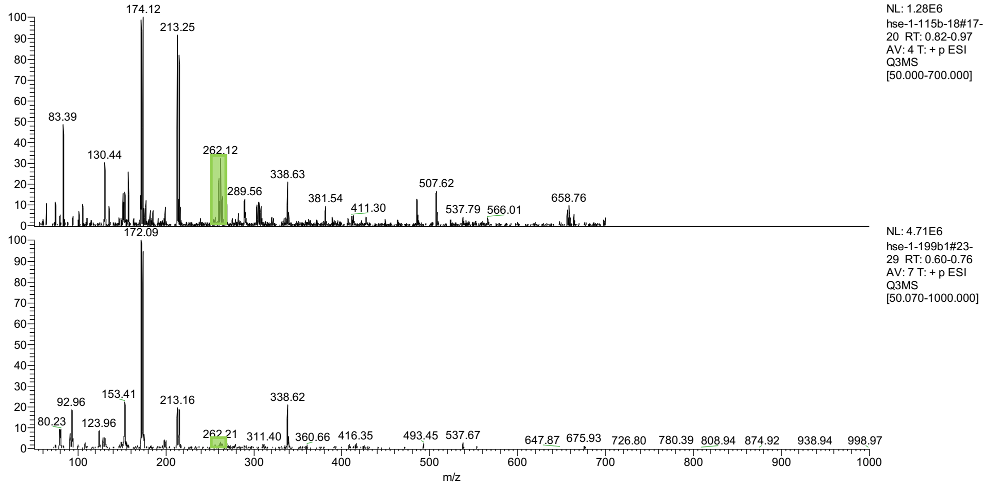

Full Scan MS of 4-methylaminebenzoate and benzyl bromide in ACN at 200 ˚C. Top T_r = 30 sec, Bottom T_r = 8 min.

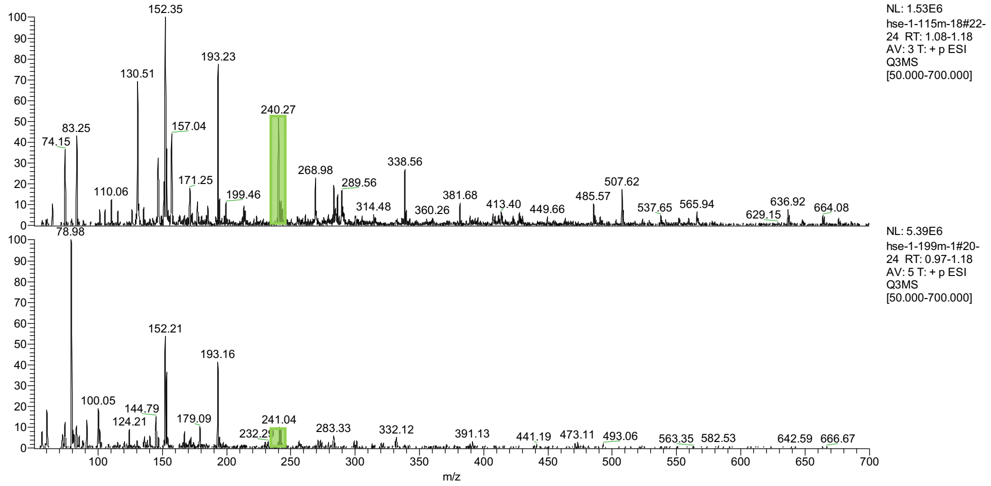

Full Scan MS of p-nitroaniline and benzyl bromide reaction in ACN at 200 ˚C. Top T_r = 30 sec, Bottom T_r = 8 min.

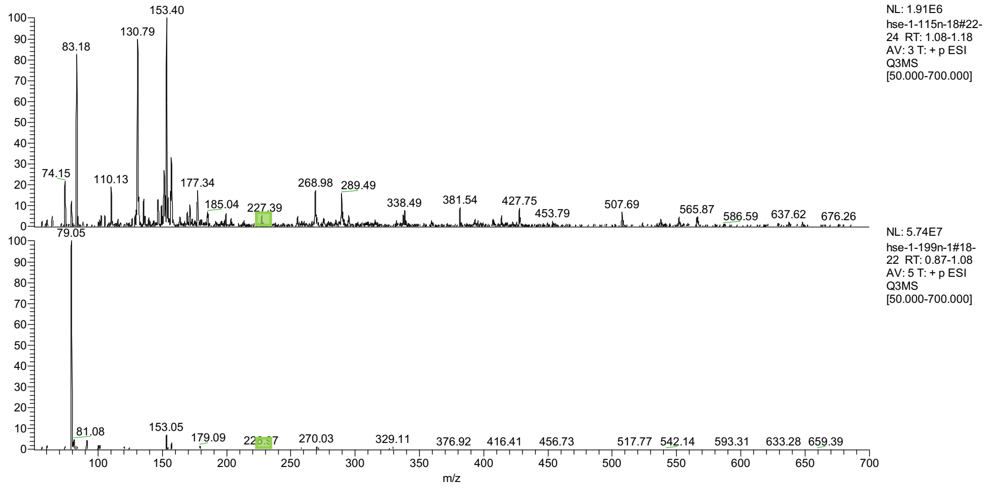

Figure S3: Expanded substrate scope experiment heat maps including ion intensity values.

DESI raw intensity (arbitrary units of ion intensity).

	Cyclohexylamine			Hexylamine			Benzylamine			Dihexylamine			Piperidine			Morpholine			N-Methylaniline			N-Methylimidazole		
Stoich.	ACN	DMSO	Tol	ACN	DMSO	Tol	ACN	DMSO	Tol	ACN	DMSO	Tol	ACN	DMSO	Tol	ACN	DMSO	Tol	ACN	DMSO	Tol	ACN	DMSO	Tol
10:1	856.4708	250.3125	3.21	992.345	252.6383	4.431667	704.3833	93.77417	3.6575	284.0325	213.6742	16.67667	215.7792	164.1183	19.52667	208.075	70.80583	16.99583	809.9783	582.5825	222.8	436.4608	247.365	134.6875
1:1	609.28	203.2875	102.3033	29.9	5.521667	3.496667	191.9058	11.7425	4.3275	32.76	48.71833	5.04	130.1158	41.63167	12.72667	278.3433	179.475	22.1475	66.905	17.5575	16.73917	49.18833	11.80083	7.704167
1:10	14.2875	9.835833	8.3025	58.89333	10.45917	6.170833	28.52417	65.42333	13.9825	75.35917	214.65	3.018333	61.3825	117.1808	5.481667	3498.087	622.7117	871.7033	2501.323	546.4692	857.3792	1982.701	207.9283	156.6233

Qualitative measurement of microflow reactions (arbitrary units of ion intensity).

		Cyclohexylamine			Hexylamine			Benzylamine			Dihexylamine			Piperidine			Morpholine			N-Methylaniline			N-Methylimidazole		
Stoich.[a]	T (°C)	ACN	DMSO	Tol	ACN	DMSO	Tol	ACN	DMSO	Tol	ACN	DMSO	Tol	ACN	DMSO	Tol	ACN	DMSO	Tol	ACN	DMSO	Tol	ACN	DMSO	Tol
10:1	50	114152.424	15471659.7	62351.4681	16691858.7	85747273.6	804322.497	101038.601	1073583.08	419786.426	58317.136	134047268	75840783.5	41175.5869	124207261	3509.08016	53533601.4	33011108.5	26484634	1078018.47	343815.022	153321.138	4655044.6	13700.6622	855952.297
1:1	50	73754.0219	413950066	107668.451	30450168.3	154782.46	807832.368	161671.022	793916.545	217674.243	303239.073	74503227.8	524330587	70498.3533	7722282.86	4468.41689	270775593	118693759	111130260	99085.4984	70612.8621	1791072.71	2990037.73	22.1004009	302460.413
1:10	50	254332.695	301156074	243501.397	114922602	166154.859	830025.887	116441.56	889045.642	22051.3493	504366.818	205431099	430056031	56622.8301	9304.31967	93912.3907	45751232.7	60356439.4	106926707	2391557.2	221378.78	569705.1	1301009.18	67.7543253	121554.468
10:1	100	113929.088	817887119	77020.0171	3147331.81	118107078	10558786.2	101956.562	1011131.77	72524.872	295119.909	155569258	75542164	37734.9196	87194508.1	77646.7864	85070353.3	64828965.6	20319920.5	2047666.75	143567.148	246153.563	13823323	182591.202	1328009.1
1:1	100	52693.1526	456507189	184736.951	2471116.26	12473610.3	10688084.1	63324.4299	540192.376	101282.836	803334.014	48433530.2	67956516.7	120362.301	109229284	275630.733	379920701	136380743	39856009.2	1578856.16	171299.182	129731.452	11747565.9	88496.565	1434189.78
1:10	100	1243755.54	154571995	66111.5349	2419625.81	28744365.4	7365582.89	98315.0836	641160.223	186520.399	269349.132	179116815	63812550.8	115419.796	128041403	2606617.1	136955199	56005126.1	45746621.8	1682891.73	341588.817	190830.656	17063597	54558.0592	994215.282
10:1	150	417716535	131962509	37760.2261	1473521	15396296.6	13703434.8	149572.137	1368249.67	64539.6444	146392.065	10811472.7	27063525.1	74960.663	130195070	324982965	188020719	66123961.7	188298295	3064510.64	269254.781	860309.273	39604982.8	4275964.61	1416173.48
1:1	150	443595778	18272707.8	53932.3168	2372379.84	47791807.2	24950908.4	71304.3852	811936.889	472335.414	574416.596	6317235.17	75575969.1	65012.0262	48218252.5	156798144	359018928	178051846	157372948	3246600.95	922208.792	583157.066	20759827.5	838809.056	1513962
1:10	150	90469956.6	51214104.6	84636.3705	2209984.46	76669634.9	17208067.2	141734.625	1038004.52	962758.069	659514.723	36606418.4	209563227	105538.65	68460067.7	119462037	248301643	78241930.1	53344499.8	2651367.44	194003.919	132578.741	71088149.8	289107.475	1121288.3
10:1	200	265935461	30243050.8	127040.369	1492087.44	30011094.1	7139168.04	60638.2094	906066.508	89322.3652	48745077	111884389	46583324.4	14644.4153	75309539.4	3215846.89	228423614	26580526.9	13733067.8	9036098	5350624.51	2076038.78	71209940.8	2781123	3791885.03
1:1	200	270459729	1374864.36	237427.498	1515200.01	32408263.9	157814.367	119639.684	231021.524	27909.1323	827998496	2618340.91	66927557.5	62659.9222	38544288	369281.046	201191478	50426059.3	4725033.28	7459802.31	2085123.44	2379671.33	96385967	3355511.13	1187404.79
1:10	200	199085356	13448052.5	265977.827	2293533.57	44974928.9	42962419.4	72621.941	621287.919	14617.0665	272637842	39105847.4	244292845	102030.634	53140603.6	173505245	102477840	31252157.1	68544878.3	8625364.74	1821724.5	615844.661	41531074.6	4427898.82	2377517.79
